# Dietary flavonoid intake and the risk of digestive tract cancers: a systematic review and meta-analysis

**DOI:** 10.1038/srep24836

**Published:** 2016-04-26

**Authors:** Yacong Bo, Jinfeng Sun, Mengmeng Wang, Jizhe Ding, Quanjun Lu, Ling Yuan

**Affiliations:** 1 Department of Nutrition and Food Hygiene, College of Public Health, Zhengzhou University, 450001 Zhengzhou, Henan, China; 2 Department of Social Medicine and Health Service Management, College of Public Health, Zhengzhou University, Zhengzhou, PR China; 3 Department of Nutrition and Food Hygiene, College of Public Health, Zhengzhou University, 450001 Zhengzhou, Henan, China; 4 Department of Nutrition and Food Hygiene, College of Public Health, Zhengzhou University, 450001 Zhengzhou, Henan, China; 5 Department of Nutrition and Food Hygiene, College of Public Health, Zhengzhou University, 450001 Zhengzhou, Henan, China; 6 Department of radiotherapy, Affiliated Tumor Hospital of Zhengzhou University, Henan Tumor Hospital, 450003 Zhengzhou, Henan, China

## Abstract

Several epidemiological studies have investigated the association between dietary flavonoid intake and digestive tract cancers risk; however, the results remain inconclusive. The aim of our study was to evaluate this association. PubMed and the Web of Knowledge were searched for relevant publications from inception to October 2015. The risk ratio (RR) or odds ratio (OR) with the 95% confidence interval (95% CI) for the highest versus the lowest categories of flavonoid intake were pooled using a fixed-effects model. A total of 15 articles reporting 23 studies were selected for the meta-analysis. In a comparison of the highest versus the lowest categories of dietary flavonoid intake, we found no significant association between flavonoid intake and oesophageal cancer (OR = 0.91, 95% CI = 0.75–1.10; I^2^ = 0.0%), colorectal cancer (OR = 1.02, 95% CI = 0.92–1.14, I^2^ = 36.2%) or gastric cancer (OR = 0.88; 95% CI = 0.74–1.04, I^2^ = 63.6%). The subgroup analysis indicated an association between higher flavonoid intake and a decreased risk of gastric cancer in the European population (OR = 0.78, 95% CI = 0.62–0.97). In conclusion, the results of this meta-analysis do not strongly support an association between dietary flavonoid intake and oesophageal or colorectal cancer. Furthermore, the subgroup analysis suggested an association between higher dietary flavonoid intake and decreased gastric cancer risk in European population.

Digestive tract cancers are very common malignant tumous worldwide and are an important cause of cancer-related death^1^,^2^. Globocan 2012 showed that the standardised incidences of colorectal cancer, gastric cancer, and oesophageal cancer placed them in the 4th, 6th, and 10th positions among all tumours, respectively^1^.

Epidemiological evidence suggests that diet may play an important role in the aetiology of digestive tract cancer risk^3^,^4^,^5^,^6^. Diets high in fruits and vegetables are inversely associated with the incidence of digestive tract cancers^5^,^6^,^7^,^8^. Flavonoids are a group of bioactive polyphenols that are abundant in plant-based foods, such as fruits and vegetables^3^. The biological effects of flavonoids for cancer prevention include the regulation of cell signaling and the cell cycle, antimutagenic and antiproliferative properties, free radical scavenging, and inhibition of angiogenesis^9^,^10^.

Several epidemiological studies have investigated the relationship between flavonoid intake and digestive tract cancer risk. However, these results are controversial. A systematic review of the literature to date might be helpful for confirming any such association. Therefore, the objective of the present study was to determine, in a meta-analysis, whether an association exists between dietary flavonoid intake and cancers of the digestive tract.

## Materials and Methods

### Search strategy

The electronic databases PubMed and Web of Knowledge (through October 2015) were searched to identify eligible studies. The following keywords were used: “flavonoids” OR “flavanones”, OR “flavones”, “anthocyanidins” OR “catechin” combined with “oesophagus cancer” OR “oesophageal squamous cell carcinoma”, “colorectal cancer” OR “colon cancer” OR “rectal cancer”; “gastric cancer” OR “stomach cancer”. Besides, we checked the reference list of all articles of interest to identify additional eligible publications.

### Study selection

The articles selected met the following criteria: (1) the studies were designed as cohort or case–control studies; (2) the exposure of interest was total dietary flavonoid intake; (3) the outcome of interest was the incidence of digestive tract cancers, including oesophageal cancer, gastric cancer, and colorectal cancer; (4) the odds ratios (ORs) or relative risk (RR) estimates with 95% confidence intervals (95% CI) were reported or could be calculated. If data were duplicated in more than one study, the one with the largest number of cases or the longest follow-up period was included in the meta-analysis.

### Data extraction

Two authors (Yacong Bo and Jinfeng Sun) independently extracted the following information from each study: the first author’s last name, year of publication, country, study design (case-control or cohort), patient characteristics (including sample size, gender, and mean age), the reported ORs (RRs) with 95% CIs for the highest versus the lowest categories of flavonoid intake, and variables adjusted in the analysis of each study. The ORs (RRs) that reflected the greatest degree of control for potential confounders were adopted in this meta-analysis. Any disagreements were resolved by a third investigator.

### Statistical analysis

The pooled ORs with 95% CIs (highest compared to the lowest category of flavonoid intake) were computed from the adjusted ORs and RRs to measure the association between dietary flavonoid intake and the risk of digestive tract cancers.

The extent of heterogeneity across studies was determined using a chi-square test and an *I*^2^ test; if *P* < 0.05 and/or *I*^2^ > 50%, indicating significant heterogeneity, a random-effect model was selected. Otherwise, a fixed-effect model was applied^11^. Meta-regression and subgroup analyses were performed to explore the possible source of heterogeneity, such as geographic region, experimental design, sample size and publication year^12^,^13^. Subgroup analyses were also performed to evaluate the potential effect of the modification of variables, including the study design, geographic region, cancer subtype, and dose. Begg’s funnel plots and Egger’s linear regression test were performed to assess the publication bias. A value of *P* < 0.05 was considered statistically significant^14^,^15^.

All analyses were conducted using STATA software (version 12.0; StatCorp, College Station, TX, USA) and a value of *P* < 0.05 was considered as statistically significant.

## Results

### Literature search and study characteristics

**Figure 1:**
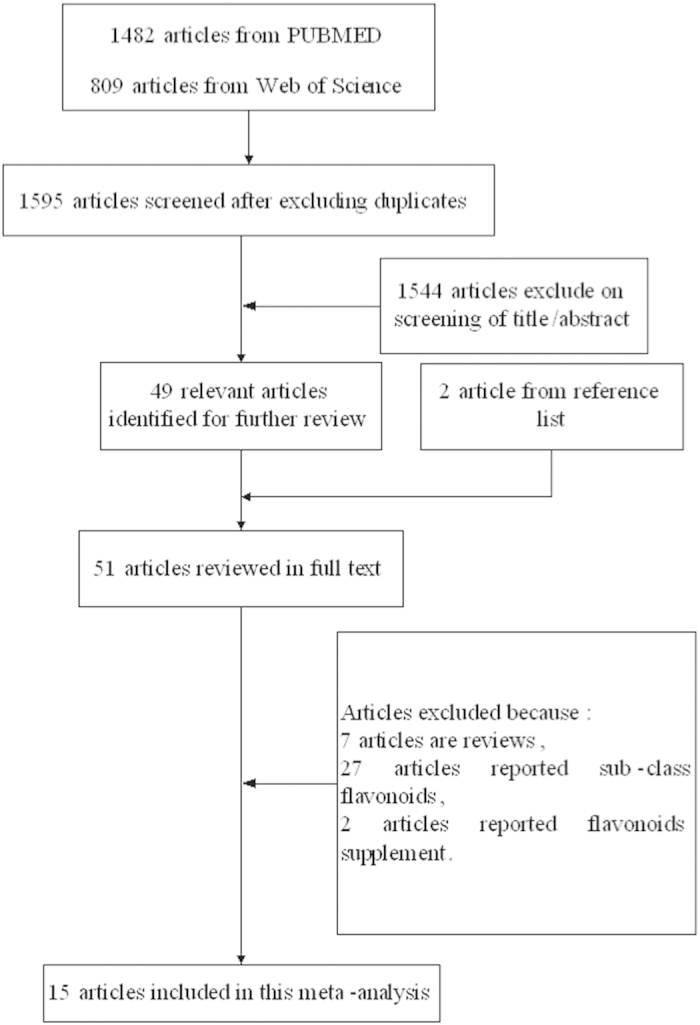
The flow diagram of screened, excluded, and analyzed publications.

The electronic search of PUBMED and web of knowledge identified a total of 1595 potentially relevant articles. Fifty-one articles were reviewed in full after reviewing the title and abstract. Among them, 7 articles were reviews, 27 articles reported sub-class flavonoids, and 2 articles reported flavonoid supplements. As a result, 15 articles reporting 23 studies, encompassing 312,734 digestive tract cancer cases and 1,142,276 controls were selected for the meta-analysis^16^,^17^,^18^,^19^,^20^,^21^,^22^,^23^,^24^,^25^,^26^,^27^,^28^,^29^,^30^. The detailed steps of our literature search are shown in Fig. 1, and the main characteristics of the included studies are shown in Table 1.

### Flavonoid intake and overall digestive tract cancer risk

**Figure 2:**
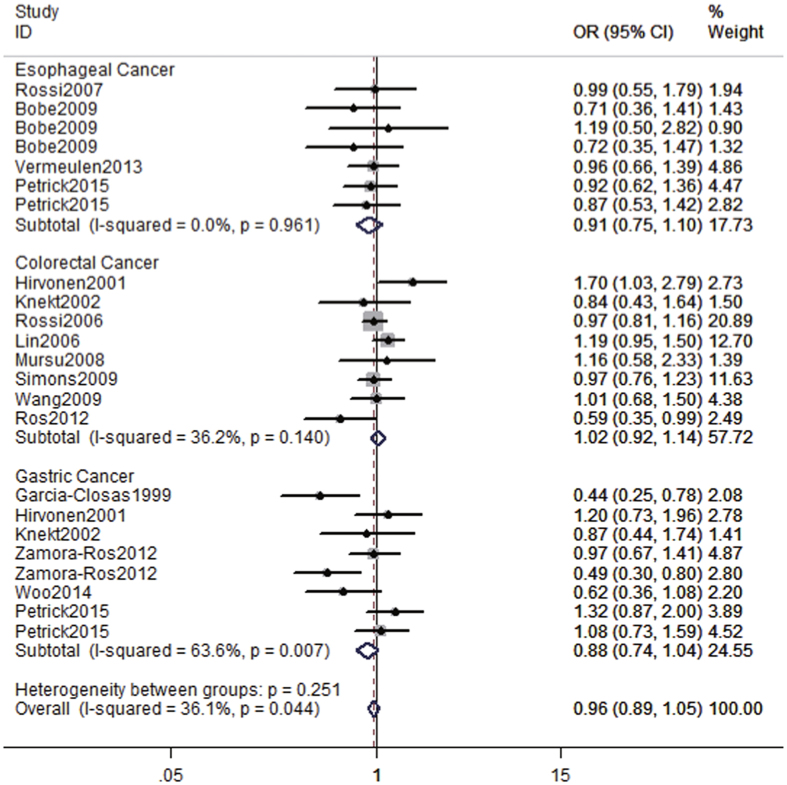
The forest plot between highest versus lowest categories of flavonoids intake and digestive tract cancers risk.

We pooled the study-specific ORs using a fixed-effect model. No significant associations were detected between the highest compared with the lowest category of flavonoid intake and digestive tract cancers (overall OR = 0.96, 95% CI = 0.89–1.05, I^2^ = 36.1%) (Fig. 2).

### Flavonoid intake and oesophageal cancer risk

Seven studies examined the association between flavonoid intake and oesophageal cancer risk. The pooled OR of oesophageal cancer for the highest versus lowest categories of flavonoid intake was 0.91 (95% CI = 0.75–1.10, I^2^ = 0.0%), suggesting that flavonoid intake was not significantly associated with the risk of oesophageal cancer (Fig. 2).

As shown in Table 2, a subgroup analysis was conducted by geographic location, study design, and histological type. However, non-significant associations of dietary flavonoid intake with oesophageal cancer were detected among all strata for the between-study subgroup analyses.

### Flavonoid intake and colorectal cancer risk

A total of eight studies assessed the association between dietary flavonoid intake and colorectal cancer risk. The pooled OR of colorectal cancer risk for the highest versus the lowest categories of flavonoid intake was 1.02 (95% CI = 0.92–1.14, I^2^ = 36.2%), indicating that flavonoid intake was not significantly associated with colorectal cancer risk.

The subgroup analysis indicated that dietary flavonoid intake has no significant effect on colorectal cancer risk in the US or European population. For colorectal cancer subtypes, no significant association was found between flavonoid intake and colon cancer (pooled OR = 0.92, 95% CI = 0.80–1.05) or rectal cancer (pooled OR = 0.90, 95% CI = 0.75–1.08), as shown in Table 2.

### Flavonoid intake and gastric cancer risk

Six studies investigated flavonoid intake and gastric cancer risk. As shown in Fig. 2, the meta-analyses demonstrated no significant association between gastric cancer risk and flavonoid intake (OR = 0.88; 95% CI = 0.74–1.04, I^2^ = 63.6%) for a comparison of the highest to the lowest category of intake.

In the subgroup analysis, we found that flavonoid intake was significantly associated with gastric cancer risk in Europe (pooled OR = 0.78, 95% CI = 0.62–0.97), but not in the United States or Asia (Table 2). With respect to study design, we did not find that flavonoid intake was statistically associated with gastric cancer in either the cohort (pooled OR = 0.86, 95% CI = 0.67–1.09) or case-control studies (pooled OR = 0.90, 95% CI = 0.72–1.13).

### Heterogeneity analysis

For most of the outcomes of digestive tract cancer, the I^2^ values of heterogeneity were lower than 50%. Only the levels of heterogeneity for gastric cancer were intermediate (I^2^ = 63.6%). To explore the sources of heterogeneity, we performed subgroup analyses with stratification by geographic location and study design. We found that flavonoid intake was significantly associated with gastric cancer risk in Europeans (pooled OR = 0.78, 95% CI = 0.62–0.97) but not in Americans or Asians. Therefore, geographic location may partially account for the appreciable heterogeneity. Meta-regression showed that study design, geographic location, source of controls, and publication year had no significant impact on heterogeneity. Moreover, the leave-one-out analysis showed that the key contributor to heterogeneity was the study conducted by Zamora-Ros^16^. After excluding this study, the heterogeneity was reduced to I^2^ = 23.4%, and the summary OR for oesophageal cancer was 1.304 (95% CI = 0.807–1.325), which was similar to the main finding.

### Publication bias

**Figure 3:**
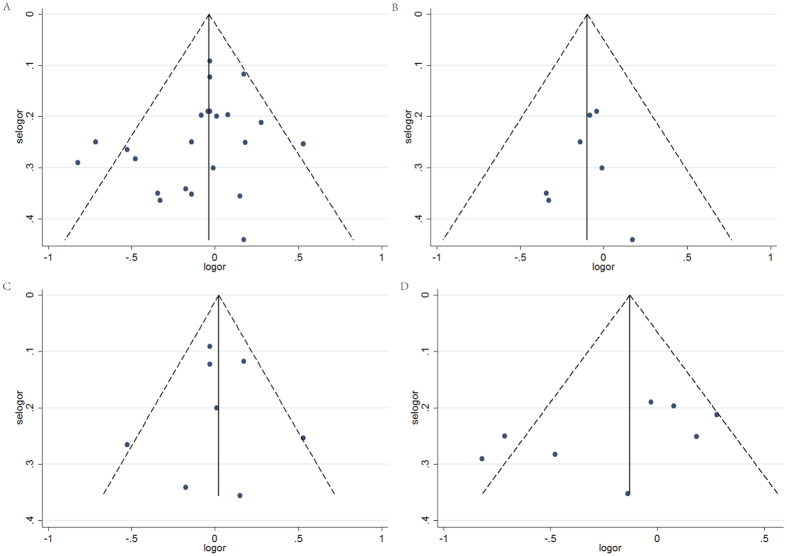
Funnel plot for publication bias of flavonoids intake and digestive tract cancers risk (**A**) Funnel plot of publication bias for all digestive tract cancers, (**B**) Funnel plot of publication bias for esophageal cancer, (**C**) Funnel plot for publication bias of colorectal cancer, (**D**) Funnel plot for publication bias of gastric cancer.

Egger’s test showed no significant publication bias in this meta-analysis (t = −1.46, *P* = 0.158 for digestive tract cancers, t = −0.49, *P* = 0.644 for oesophageal cancer, t = −0.06, *P* = 0.951 for colorectal cancer, and t = −1.56, *P* = 0.170 for gastric cancer), and the funnel plots are shown in Fig. 3.

## Discussion

The present meta-analysis first evaluated the association between dietary flavonoid intake and the risk of digestive tract cancers based on the highest versus the lowest categories. We found no significant association between the highest dietary flavonoid intake and oesophageal cancer, colorectal cancer, or gastric cancer. However, studies conducted in European populations indicated an association between higher flavonoid intake and a decreased risk of gastric cancer.

Previous epidemiological studies have suggested that flavonoid intake has no significant effect on breast cancer (RR = 0.98, 95% CI = 0.86–1.12)^31^ or colorectal neoplasms (RR = 1.03, 95% CI = 0.88–1.20 )^32^. The meta-analysis also suggested a significant association between the highest category of flavonoid intake and a reduced risk of lung cancer (RR = 0.76, 95% CI = 0.63–0.92)^33^. The association between dietary flavonoid intake and the risk of digestive cancers identified in our meta-analysis adds new information regarding the relationship between flavonoid intake and cancer risk.

The lack of a strong association between flavonoid intake and oesophageal or colorectal cancer is particularly surprising because numerous *in vitro* and animal studies have demonstrated an inverse association between flavonoid and oesophageal or colorectal cancer^34^,^35^,^36^,^37^. However, many flavonoids present in foods cannot be absorbed in their native form, including esters, glycosides, and polymers^38^. It has been demonstrated that the amount of flavonoids that are bioavailable is only a small proportion of the ingested amount, ranging from 0.2–0.9% for tea catechins to 20% for quercetin and isoflavones^39^,^40^. In *in vitro* and animal studies, the intake of flavonoids is much higher than that in humans, and it remains unclear whether the beneficial antiproliferative and antioxidative effects observed during *vitro* studies are also present in humans^41^. Other studies have also suggested that flavonoids have weaker actions *in vivo* than *in vitro*^42^.

In the subgroup analysis by geographic location, we found an inverse association between flavonoid intake and gastric cancer in Europe but not in America or Asia. The reasons for this may include the complexity due to the presence of flavonoids from various food sources, the occurrence of a large amount of flavonoids in nature, and the diversity of dietary culture^43^. The main dietary sources of flavonoids are fruits, vegetables, tea, and red wine^44^. However, the flavonoids in vegetables and fruits depend on the type of cultivation, crop variety and location, as well as the specific morphological part of the plant^45^. Moreover, we could not exclude cultural differences in the storage and preparation of foods, particularly vegetables, which may also lead to this result.

Medium heterogeneity was detected for the association between flavonoid intake and gastric cancer. A meta-regression was used to explore the sources of heterogeneity, which showed that the study design, geographic location, source of controls, and publication year had no significant impact on heterogeneity. Then, we performed subgroup analyses by study design and geographic location and, found that flavonoid intake was significantly associated with gastric cancer risk in Europe, but not in America or Asia, suggesting that the intermediate heterogeneity is partially explained by geographic location. Moreover, the leave-one-out analysis showed that the key contributor to heterogeneity was the study conducted by Zamora-Ros^16^. After excluding this study, the summary estimate was not materially altered, but the I^2^ of heterogeneity was reduced from 63.6% to 23.4%.

The role of flavonoids in gastric carcinogenesis has been attributed to several mechanisms. Flavonoids have anticarcinogenic effects by way of their antioxidant properties, which are attributed to their ability to modulate antioxidant pathways^46^. Moreover, flavonoids can regulate cell proliferation and apoptosis, modulate phase I and II metabolic enzymes, and affect inflammatory pathways^47^. Another possible explanation for the protective effects of flavonoids m may be their potential anti-Helicobacter pylori effect^48^, including direct bactericidal activity, the neutralisation of VacA, the reduction of urease secretion, and interference with Toll-like receptor 4 signaling^49^, which is unique to gastric cancer. Therefore, high dietary flavonoids intakes can possibly reduce the risk of gastric but not esophageal or colorectal cancer.

Several potential limitations of our meta-analyses should be acknowledged. The relatively small number of studies analysed makes it difficult to evaluate heterogeneity. Furthermore, we only compared the risk of cancer for those in the highest category of flavonoid intake with that of those in the lowest category of flavonoid intake, and the definition of these intake categories varied between studies. In addition, only published studies were included in the meta-analysis, which may have biased the results.

## Conclusion

In summary, this meta-analysis provides little evidence of an association between the highest category of dietary flavonoid intake and the risk of oesophageal cancer or colorectal cancer. However, a subgroup analysis demonstrated that dietary flavonoids could reduce the risk of gastric cancer in the European population although not the U.S. or Asian populations.

## Additional Information

**How to cite this article**: Bo, Y. *et al.* Dietary flavonoid intake and the risk of digestive tract cancers: a systematic review and meta-analysis. *Sci. Rep.* **6**, 24836; doi: 10.1038/srep24836 (2016).

## Figures and Tables

**Table 1:** Characteristics of studies on flavonoids intake and digestive tract cancers risk.

First author, year	Country	Cancer site	Study design	Dietary assessment	Participants (cases)	Intake comparison, High vs. low (mg/d)	RR (95% CI) for highest vs lowest category	Adjustment for covariates
Bobe^27^	America	EC	Case–control	75-item FFQ	2406 (493)	White: >107 vs < 43.0 Black > 99.6 vs < 38.6	White-EAC :0.71 (0.36–1.42), White-ESCC: 1.19 (0.50–2.81) Black-ESCC: 0.72 (0.35–1.46)	smoking duration and intensity, geographical area, age, body mass index, hot tea consumption, hard liquor consumption, beer consumption, red wine consumption, white wine consumption
Vermeulen^26^	European	EC	Cohort	Validated FFQ	477312 (341)	NR	0.96 (0.66–1.39)	center, age, sex, energy intake, body mass index, smoking intensity, educational level, physical activity, alcohol, red and processed meat, fiber, vitamin C, and carotenoids intake.
Petrick^28^	America	EC and GC	Case–control	104-item FFQ	1716 (EC:465, GC: 589)	≥217.36 vs. 0–63.81	EAC:0.92 (0.63–1.37) ESCC: 0.87 (0.53–1.41) GCA: 1.32 (0.87- 2.00) OGA: 1.08 (0.73, 1.58)	age, sex, race, geographic centre, cigarette smoking, and dietary energy intake
Rossi^25^	Italy	EC	Case–control	78-item FFQ	107 (304)	>217.4vs < 96.5	0.99 (0.55–1.79)	age, sex, study centre, education, alcohol consumption, tobacco smoking, body mass index and energy intake
Hirvonen^19^	Finland	CC and GC	Cohort	276-item FFQ	25776 (CC: 133, GC: 111)	16.3 vs. 4.2 (median)	CC: 1.7 (1.0–2.7) GC: 1.2 (0.71–1.9)	age and supplementation group
Rossi^24^	Italy	CC	Case–control	78-item FFQ	6107 (1953)	>191.1vs < 75.3	0.97 (0.81–1.16)	age, sex, study center, family history of colorectal cancer, education, alcohol consumption, body mass index, occupational physical activity, and energy intake, according to the residual model
Zamora-Ros^23^	Spain	CC	Case–control	600-item FFQ	825 (424)	>167.9vs < 68.9	0.59 (0.35–0.99)	sex, age, BMI, energy intake, alcohol and fiber intake, red and processed meat intake, tobacco consumption, physical activity, regular drugs, and family history of colorectal cancer
Lin^22^	America	CC	Cohort	131-item FFQ	107401 (878)	Nurses’ Health Study cohort: >31.1 vs < 9.6 Health Professionals Follow-up Study cohort: >30.5 vs < 10.7	1.19 (0.94–1.49)	djusted for age, body mass index, family history of colorectal cancer, history of colorectal polyps, prior sigmoidoscopy screening, physical activity, smoking status, red meat intake, alcohol consumption, total energy, calcium, folate, and fiber intake, aspirin use, and multivitamin use
Mursu^21^	Finland	CC	Cohort	4-day food recording	2590 (55)	NR	1.16 (0.58–2.34)	age and examination years, BMI, smoking status, pack-years of smoking, physical activity, alcohol, total fat, saturated fat, energy adjusted intake of fiber, vitamin C and E.
Simons^20^	Netherland	CC	Cohort	150-item FFQ	3906 (2219)	36.0–105.0 vs. 1.4–16.0	0.97 (0.76–1.23)	age, family history of colorectal cancer, smoking status, alcohol intake, nonoccupational physical activity, BMI and processed meat intake
Wang^17^	America	CC	Cohort	131-item FFQ	38408 (3234)	34.55–236.38 vs. 0–11.55	0.93 (0.83–1.03)	Age, race, total energy intake, randomized treatment assignment, smoking, alcohol use, physical activity, postmenopausal status, hormone replacement therapy use, multivitamin use, BMI, family history of colorectal cancer, ovary cancer, and breast cancer, and intake of fruit and vegetables, fiber, folate, and saturated fat
Knekt^30^	Finnish	CC and GC	Cohort	>100-item FFQ	10054 (CC: 90, GC: 74)	NR	CC: 0.84 (0.43, 1.64) GC: 0.87 (0.44, 1.75)	sex, age, geographic area, occupation, smoking, and BMI
Garcia-Closas^29^	Spain	GC	Case–control	77-item FFQ	708 (354)	NR	0.44 (0.25–0.78)	intake of nitrites, nitrosamines, vitamin C, total energy, and total carotenoids
Woo^18^	Korea	GC	Case–control	103-item FFQ	668 (334)	152.3 vs. 52.5 (median)	0.62 (0.36–1.09)	Total energy intake, H. pylori, age, sex, education, smoking status, alcohol consumption, BMI, physical activity, and consumption of pickled vegetable and red and processed meat, fruits and vegetable consumption.
Zamora-Ros^16^	European	GC	Cohort	Validated FFQ	477386 (683)	>595.5 vs < 200.4	0.97 (0.67–1.41) for men, 0.49 (0.30–0.80) for women	center, age, and sex and adjusted for energy intake, body mass index, smoking intensity, educational level, physical activity, alcohol, and red and processed meat intake, fiber, vitamin C, and carotenoids

Abbreviations: FFQ, Food Frequency Questionnaire; OR, Odds Ratio; CI, Confidence Interval; GCA, Gastric Cardia Adenocarcinoma; OGA, Other Gastric Adenocarcinoma; EAC, Esophageal Adenocarcinoma; ESCC, Esophageal Squamous Cell Cancer, CC, Colorectal cancer; GC, Gastric Cancer.

**Table 2:** Subgroup analysis of flavonoids intake and risk of esophageal, colocteral and gastric cancer.

Subgroups	Cancer site	No. ofcases	No. ofstudies	OR (95% CI)	Heterogeneity test	
					*I*^2^ (%)	*P*
Geographic locations						
Europe	Esophageal	645	2	0.97 (0.71–1.33)	0	0.931
America	Esophageal	4558	5	0.87 (0.68–1.11)	0	0.879
Cancer subtype						
ESCC	Esophageal	827	4	0.91 (0.66–1.24)	0	0.827
EAC	Esophageal	435	2	0.86 (0.62–1.21)	0	0.520
Study design						
Cohort	Esophageal	341	1	0.96 (0.66–1.39)	N/A	N/A
Case-control	Esophageal	1262	6	0.89 (0.71–1.11)	0	0.930
Geographic locations						
Europe	Colorectal	4847	7	0.96 (0.86–1.08)	34.5	0.165
America	Colorectal	4112	2	1.14 (0.94–1.39)	0	0.480
Cancer subtype						
Colon	Colorectal	3005	4	0.92 (0.80–1.05)	46.6	0.132
Rectum	Colorectal	1388	4	0.90 (0.75–1.08)	0	0.482
Study design						
Cohort	Colorectal	6609	7	1.05 (0.93–1.19)	16	0.308
Case-control	Colorectal	2377	2	0.92 (0.78–1.09)	68.1	0.076
Geographic locations						
Europe	Gastric	1222	5	0.78 (0.62–0.97)	65.8	0.020
America	Gastric	589	2	1.19 (0.89–1.57)	0	0.488
Asia	Gastric	334	1	0.62 (0.38–1.08)	N/A	N/A
Study design						
Cohort	Gastric	1277	4	0.86 (0.67–1.09)	74.8	0.008
Case-control	Gastric	868	4	090 (0.72–1.13)	58.4	0.065

Abbreviations: OR, Odds Ratio; EAC, Esophageal Adenocarcinoma; ESCC, Esophageal Squamous Cell Cancer.
